# Stomata Prioritize Their Responses to Multiple Biotic and Abiotic Signal Inputs

**DOI:** 10.1371/journal.pone.0101587

**Published:** 2014-07-08

**Authors:** Xiaobin Ou, Yi Gan, Peilei Chen, Muqing Qiu, Kun Jiang, Genxuan Wang

**Affiliations:** 1 College of Life Sciences, Zhejiang University, Hangzhou, China; 2 School of Agriculture and Food Sciences, Zhejiang A&F University, Hangzhou, P. R. China; 3 School of Life Sciences, Shaoxing University, Shaoxing, P. R. China; Key Laboratory of Horticultural Plant Biology (MOE), China

## Abstract

Stomata are microscopic pores in leaf epidermis that regulate gas exchange between plants and the environment. Being natural openings on the leaf surface, stomata also serve as ports for the invasion of foliar pathogenic bacteria. Each stomatal pore is enclosed by a pair of guard cells that are able to sense a wide spectrum of biotic and abiotic stresses and respond by precisely adjusting the pore width. However, it is not clear whether stomatal responses to simultaneously imposed biotic and abiotic signals are mutually dependent on each other. Here we show that a genetically engineered *Escherichia coli* strain DH5α could trigger stomatal closure in *Vicia faba*, an innate immune response that might depend on NADPH oxidase-mediated ROS burst. DH5α-induced stomatal closure could be abolished or disguised under certain environmental conditions like low [CO_2_], darkness, and drought, etc. Foliar spraying of high concentrations of ABA could reduce stomatal aperture in high humidity-treated faba bean plants. Consistently, the aggressive multiplication of DH5α bacteria in *Vicia faba* leaves under high humidity could be alleviated by exogenous application of ABA. Our data suggest that a successful colonization of bacteria on the leaf surface is correlated with stomatal aperture regulation by a specific set of environmental factors.

## Introduction

Stomata are natural tiny openings on leaf surface that regulate intake of CO_2_ for photosynthesis and water loss through transpiration. Active stomatal aperture regulation is one of the earliest plant adaptive traits against adverse environmental changes and therefore has been intensively investigated [1]–[3]. Stomata can respond to a wide array of environmental stimuli. Specifically, they open under light and high relative humidity (RH) conditions, while close when treated with darkness, low RH, drought or the stress-responsive phytohormone ABA [4]–[8].

A plethora of signaling components have been identified in environmental stimulus-triggered stomatal movement [1], [5]. Light promotes stomatal opening by activating a set of light receptors on the guard cell surface and subsequently increase the activity of proton pumps (H^+^-ATPases) coupled to them [9]–[11]. The massive efflux of H^+^ leads to the compensative influx of potassium ions into guard cells, which ultimately induce stomatal opening [12], [13]. Light-induced stomatal opening can be inhibited by ABA, while ABA *per se* can trigger closure of the stomatal pore [14]. The signal transduction network of ABA in guard cells encompasses signaling events like depolarization of plasma membrane, cytosolic calcium oscillation, production of ROS, protein modification, actin reassembly, and gene expression [15]–[21]. Unlike the above extrinsic signals that tend to show unilateral effect on stomatal aperture, some other environmental factors display dual effects that are relevant to their doses. For example, high concentrations of CO_2_ (>800 ppm) induce rapid stomatal closure, a process that involves the function of two *β*-carbonic anhydrases (*β*-CA1 and *β*-CA4), a guard cell-specific protein kinase (HT1), and a plasma membrane-localized ABC transporter (AtABCB14) [22]–[24]. In contrast, low concentrations of CO_2_ (<100 ppm) trigger stomatal opening, although the underlying mechanism remains largely unknown. The effect of RH on stomatal aperture also depends on its value, i.e. high RH (>90%) promotes stomatal opening whilst low RH (<25%) causes stomatal closure. Interestingly, both of the above processes are mediated by ABA signaling in guard cells [4], [8], [25].

Recent studies also suggest that stomata function as the first barrier of plants against invasion of foliar bacterial pathogens [26]–[28]. It was found that stomata closed rapidly when leaves were inoculated with human or plant pathogenic bacteria [26], [27]. Such a response helps to cut off the entry of foliar pathogens into leaf inner space during the first contact. Interestingly, plant pathogenic bacteria like *Pseudomonas syringae* pv tomato DC3000 have developed mechanisms to reopen the stomata pore by secreting specific virulent factors [27]. Rapid stomatal closure was initiated from the recognition of pathogen associated molecular patterns (PAMPs) by specific membrane receptors, such as FLS2 [27], [29]. *flg22*, a PAMP derived from bacterial flagellin, inhibits light-induced stomatal opening by inhibiting the inward K^+^ channels, but such a response is diminished in the null mutant of the heterotrimeric G-protein α subunit GPA1 [30]. Moreover, it was reported that the mitogen-activated protein kinase 3 (MPK3) is involved in the bacteria or lipopolysaccharide-induced stomatal closure in *Arabidopsis* [26], while MPK9 and MPK12 are involved in chitosan-induced stomatal closure [31]. FIN4, which encodes a chloroplastic enzyme ASPARTATE OXIDASE, is responsible for the PAMP-triggered, RBOHD-dependent ROS burst and stomatal immunity [32]. ABA and SA signaling events are also involved in stomatal immune response. It has been found that *Arabidopsis* ABA mutants *ost1* and *aba3* showed impaired stomatal response to *Pst* DC3000, suggesting an indispensible role of ABA in the stomatal defense against foliar bacterial pathogens [27]. Moreover, a recent screening in *Arabidopsis* recovered several mutants (*scord1, scord3, scord5, scord6* and *scord8*) that are compromised in stomatal immunity but show wild-type responses to ABA, pointing to an additional, ABA-independent mechanism that underlies stomata-based defense [29].

There is a growing body of evidence suggesting that plant defense against pathogenic microorganisms can be altered under certain environmental conditions. For instance, plants are more vulnerable to bacterial pathogen attack after heavy rain or under high RH conditions [33]. Invasion of the human pathogen *Salmonella enterica* into lettuce leaves is more severe under light [34]. By contrast, osmotic stresses could increase plant resistance to *Botrytis cinerea* and *Oidiumneoly copersici* [35]. In a study performed under elevated [CO_2_] for three consecutive years, the disease incidence and severity of fungal pathogen *Phyllosticta minima* were significantly reduced in *Acer rubrum* [36]. All the evidence above implies that environmental perturbation is closely associated with the interaction between plants and microbes.

Although the correlation between environmental regulation of stomatal aperture and plant defense against pathogenic microbes has been proposed for decades, there are only few studies focusing on the behavior of stomata exposed simultaneously to biotic and abiotic stimuli. Moreover, most of the existing studies were performed on the model plant *Arabidopsis*. In this work, we exploited two complementary approaches, i.e. epidermal bioassay and gas exchange measurement, to examine stomatal responses to multiple extrinsic signals in *Vicia faba* as well as in *Arabidopsis*. We found that stomatal closure triggered by DH5α could be abolished by low [CO_2_] and high RH, but disguised by darkness and root water deficiency. We also demonstrated that multiplication of DH5α in *V. faba* leaves could be reduced significantly by exogenous ABA under high RH conditions. Our data suggest that there is a prioritization among stomatal responses to simultaneous biotic and abiotic signal inputs.

## Materials and Methods

### Plant materials

Both faba bean (*Vicia faba* L.) and *Arabidopsis* (*Arabidopsis thaliana*, ecotype Col-0) plants were used in this study. *V. faba* plants were grown in a greenhouse with controlled temperature (22°C–26°C), a relative humidity of 70%, and a 14/10 light regime under light intensity of 400 µmol/m^2^/s. Fully expanded leaves of 4-week-old plants were chosen for all the experiments. *Arabidopsis* plants were grown in 6 cm×6 cm square plastic pots filled with Fafard growing mix (Canada) in a controlled growth chamber at 22°C with a 10/14 photoperiod under light intensity of 120 µmol/m^2^/s. For all the experiments requiring *Arabidopsis*, 4- to 5-week-old plants were used.

### Bacterial strains and inoculation

The *E. coli* strain DH5α was cultured in Luria-Bertani (LB) broth at 37°C with agitation at 220 rpm for 10 hours. Bacterial cells were collected by centrifugation at 6000×g for 30 minutes and then re-suspended in sterilized water. The *Pseudomonas syringae* strain pv tomato DC3118 (*Pst* DC3118) were cultured at 30°C in LB medium supplemented with appropriate antibiotics [27]. Plants were inoculated by dipping fully expanded rosette leaves into bacterial suspension (10^8^ CFU/ml) for 15 seconds.

### Setting of environmental conditions

To evaluate the influence of various extrinsic stimuli on stomatal immunity, an informative set of environmental factors either promoting stomatal opening or closure was chosen for testing. To examine the effect of low [CO_2_] on stomatal immunity, air was pumped into an airtight plant growth chamber (1 m×0.5 m×0.6 m) following filtration through a tube containing CO_2_ absorbent. The CO_2_ concentration inside the chamber was monitored by a handheld leaf photosynthesis system (CI-340, CID, USA). For the RH experiment, an air humidifier (YADU, China) was used to manipulate the relative air humidity in the growth chamber. The RH value in the chamber was determined using a portable RH monitor (Testo 625, Germany). In the light/dark transition experiment, inoculated *V. faba* plants were incubated either under illumination (400 µmol/m^2^/s) or in darkness for one hour before stomatal measurements were performed. In the root water deficiency experiment, the field water content (FWC) was manipulated by withdrawing daily irrigation. The FWC value was determined using a soil moisture meter (HH2 with WET1 sensor, Delt-T Devices Ltd., UK). Inoculation was performed when the FWC dropped to designate values.

### Assessment of stomatal responses to treatments

Stomatal responses were assessed using two complimentary assays. To assess the responses of stomata in epidermal peels, fully expanded rosette leaves were inoculated with mock or bacterial suspension (10^8^ CFU/ml) and then plants immediately moved back to the growth chamber with indicated environmental settings. After one hour, epidermal peels were peeled from the abaxial side of leaves and observed under the microscope (Nikon E600, Japan). Forty randomly selected stomata were photographed and the aperture was measured with the Image Pro-Plus software (Media Cybernetics Co., USA). To determine gas exchange *in planta*, stomatal conductance (Gs) was recorded by the CI-340 portable leaf photosynthesis systems (CID, USA) at the light intensity of 400 µmol/m^2^/s.

### Determination of ROS levels in guard cells

The accumulation of ROS in guard cells was determined by using the fluorescent dye H_2_DCF-DA (Invitrogen, USA) as described previously [19]. The abaxial epidermal peels of fully expanded leaves from 4-week-old *V. faba* plants were floated on the opening buffer containing 50 mM KCl and 10 mM MES-Tris (pH 6.15) for 3 h under light (120 µmol/m^2^/s) to pre-open stomata. The peels were then transferred to the loading buffer containing 50 µM H_2_DCF-DA and incubated in the dark for 10 min. The fluorescent dye-loaded peels were washed 3 times with the opening buffer to remove excess dye from the apoplast, which was precedent to the incubation of them in the LPS solution (200 ng/µl) or in the DH5α suspension (10^8^ CFU/ml) for 15 min. To investigate the possible involvement of NADPH oxidases in DH5α-elicited stomatal closure, the DPI solution (20 µM) was added to the opening buffer 30 min before dye loading.

Examination of fluorescence was performed using an inverted laser-scanning confocal microscope (LSM510, Zeiss, Germany). The field of vision was photographed under 488/520 nm of elicitation/absorption. The average fluorescent intensity of 20–30 stomata were measured and calculated with the Image-Pro Plus software (USA).

### Assays on bacterial inoculation

*Vicia faba* plants were acclimated in a growth chamber under ambient (60±5%) or high (≥90%) RH for 12 hours before dip-inoculated with DH5α cell suspensions (10^8^ CFU/ml) or the mock solution supplemented with 0.02% Silwet-L77. For experiments requiring exogenous ABA, an ABA stock solution (10 mM) was added directly into each inoculum to the final concentration of 10 or 20 µM. Inoculated plants were immediately moved back to the growth chamber with RH settings the plants acclimated to. Leaf samples were collected after 3 days and bacterial enumeration was performed as previously described [27].

The progression of bacterial disease symptoms in *Arabidopsis* upon DC3118 inoculation was assessed according to a previous report [27]. Plants pretreated with ambient (60±5%) or high (≥90%) RH were dip-inoculated with mock or DC3118 suspension (10^8^ CFU/ml) and then grown under the indicated RH for 4 days. To examine the effect of ABA on disease symptom progression, leaves were sprayed with ABA (20 µM) thirty minutes before inoculation, and the same concentration of ABA was sprayed once a day in the next 4 days.

### Statistical analysis

All the experiments were repeated at least 3 times. The data were analyzed by Student's two-tailed *t*-test at p≤0.05 using SPSS 12.0 (SPSS Inc. USA).

## Results

### Stomatal closure induced by DH5α is associated with ROS accumulation in *Vicia faba*

**Figure 1:**
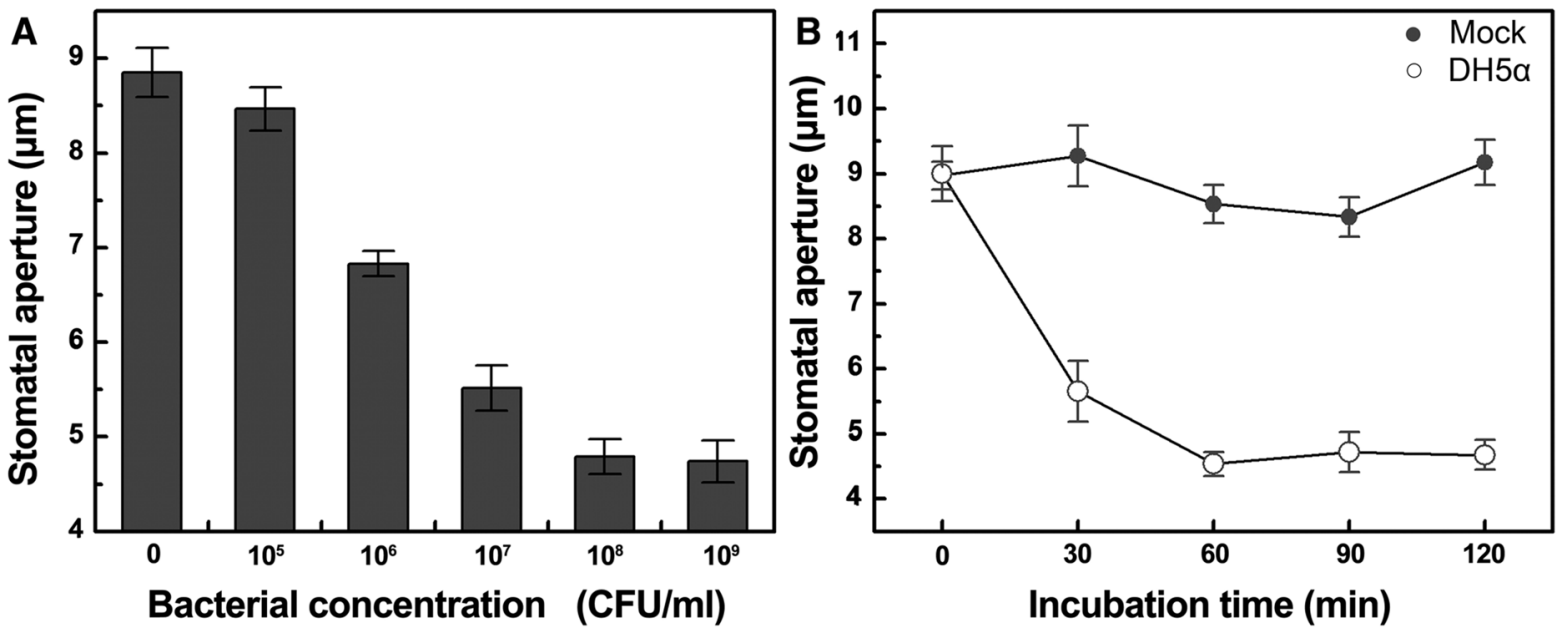
*E. coli* DH5α can trigger stomatal closure in *V. faba*. A, Stomatal aperture in *V. faba* epidermal peels incubated with DH5α at the indicated concentrations; B, Stomatal aperture in *V. faba* epidermal peels incubated with mock or DH5α at 10^8^ CFU/ml. Results represent means of three replicates ±SEM, (n = 120 stomata).

Previous studies have shown that stomata close promptly when coming in contact with microorganisms, a plant defensive response known as stomatal immunity [37]–[39]. Pathogenic bacteria like *Pseudomonas syringae* DC3000 can bypass such a pre-immune response by secreting virulence factor coronatine (COR) that will ultimately cause reopening of the stomatal pore [27]. Since we were about to investigate the putative effects of environmental factors on stomatal immunity, we started from looking for bacterial strains that would only lead to stomatal closure. Using genetically engineered bacteria *E. coli* DH5α (shortened as DH5α hereafter) to dip inoculate faba bean leaves, we found that this bacterial strain could induce rapid and sustained closure of the stomatal pore in a dose- and time-dependent manner (Figure 1A and 1B). The reduction in stomatal aperture triggered by DH5α at the concentration of 10^8^ CFU/ml was comparable to that induced by a higher level (about 10-fold) of DH5α cell suspension (Figure 1A). When inoculating *Vicia faba* leaves with DC3118, a coronatine-deficient mutant of *Pseudomonas syringae* DC3000, we observed a similar pattern of stomatal immune response (Figure S1). Considering the simplicity of manipulating DH5α under lab conditions, we performed the rest of our experiments with DH5α inoculum at the concentration of 10^8^ CFU/ml.

**Figure 2:**
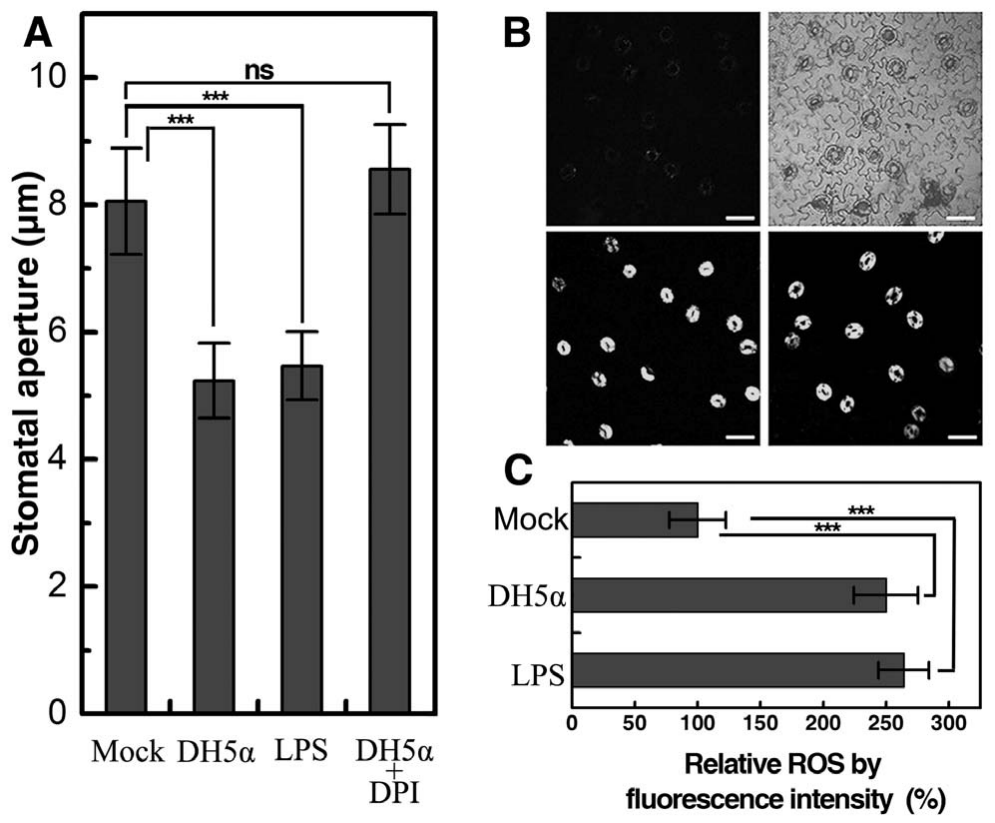
DH5α-induced stomatal closure involves ROS accumulation in guard cells. A, Stomatal aperture in *V. faba* epidermal peels incubated with different treatments. Data are means of 120 stomatal aperture measurements from three replicates ±SEM; B, ROS accumulation in intact guard cells detected by H_2_DCF-DA fluorescence. The microscopic images represent fluorescent and DIC images of peels treated with mock (upper left and right), fluorescent image of peels treated with DH5α at 10^8^ CFU/ml (lower left), and fluorescent image of peels treated with 1 mM LPS (lower right). Bars  =  50 µm; C, Quantitation of generated ROS in *Vicia faba* guard cells as shown in B. Asterisks denote significant differences as analyzed by two-tailed *t*-test (***, *P*<0.001; ns, no statistical difference).

Generation of reactive oxygen species (ROS) is an early signaling event in ABA-induced stomatal closure [19], [21]. ROS generation has also been found to mediate stomatal immune response [40]. To test whether ROS burst was involved in DH5α-induced stomatal closure, we took a pharmacological approach. It was found that both DH5α and a bacterial elicitor LPS [41] could cause stomatal closure within an hour, whilst these responses were completely abolished by pretreatment of epidermal peels with 20 µM diphenylene iodonium (DPI), a non-specific inhibitor of NADPH oxidase (Figure 2A). These data suggest that intracellular ROS accumulation is critical for DH5α- or LPS-triggered stomatal closure in *Vicia faba*. To provide direct evidence for the involvement of ROS generation in such a stomatal immune response, we measured the levels of hydrogen peroxide in guard cells from different treatments. There was a significant increase (*P*<0.001) in the green fluorescence intensity in peels treated with *E. coli* suspensions (10^8^ CFU/ml) or LPS (200 ng/µl) (Figure 2B and 2C). In consistence with the epidermal bioassay data, ROS burst was completely abolished by DPI pretreatment (Figure S2).

### A low concentration of CO_2_ and high relative humidity can abolish DH5α-induced stomatal closure

**Figure 3:**
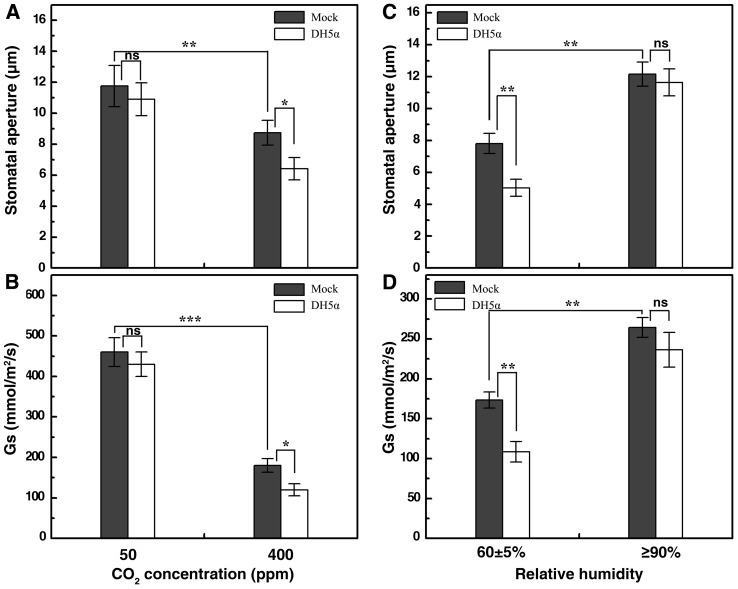
DH5α-elicited stomatal closure is abolished by low [CO_2_] or high RH treatment. A and B, Stomatal aperture and conductance in *V. faba* leaves dip-inoculated with mock or DH5α at 10^8^ CFU/ml under ambient or low CO_2_ concentrations; C and D, Stomatal aperture and conductance in *V. faba* leaves dip-inoculated with mock or DH5α at 10^8^ CFU/ml under ambient and high RH conditions. Data from the epidermal bioassay are means of 120 stomatal aperture measurements from three replicates ±SEM. Data from the stomatal conductance experiment are means of measurements from 8–12 leaves (n = 4). Asterisks denote significant differences as analyzed by two-tailed t-test (***, P<0.001; **, P<0.01; *, P<0.05; ns, no statistical difference).

Since DH5α could induce rapid and sustained stomatal closure in *Vicia faba*, we exploited it as our experimental platform to examine the effects of environmental stimuli on stomatal immune response. We chose low [CO_2_] and high RH as representative environmental signals that lead to stomatal opening [1], [6]. Plants were surface inoculated with DH5α suspensions and then placed under the indicated CO_2_ concentrations or RH conditions. Stomatal aperture and leaf gas exchange were measured one hour later. We found that DH5α could trigger stomatal closure under ambient CO_2_ concentration (400 ppm) but displayed no discernible effect on stomatal aperture when the CO_2_ concentration was reduced to 50 ppm (Figure 3A and 3B). Similarly, DH5α caused significant decrease in stomata aperture and Gs (27% and 38%, respectively) under ambient RH, whilst such a response was completely abolished when the RH increased to above 90% (Figure 3C and 3D). These results suggest that stomatal opening signals like a low concentration of CO_2_ or high RH can override stomatal closure induced by DH5α bacteria.

### Darkness and drought disguise DH5α-induced stomatal closure

**Figure 4:**
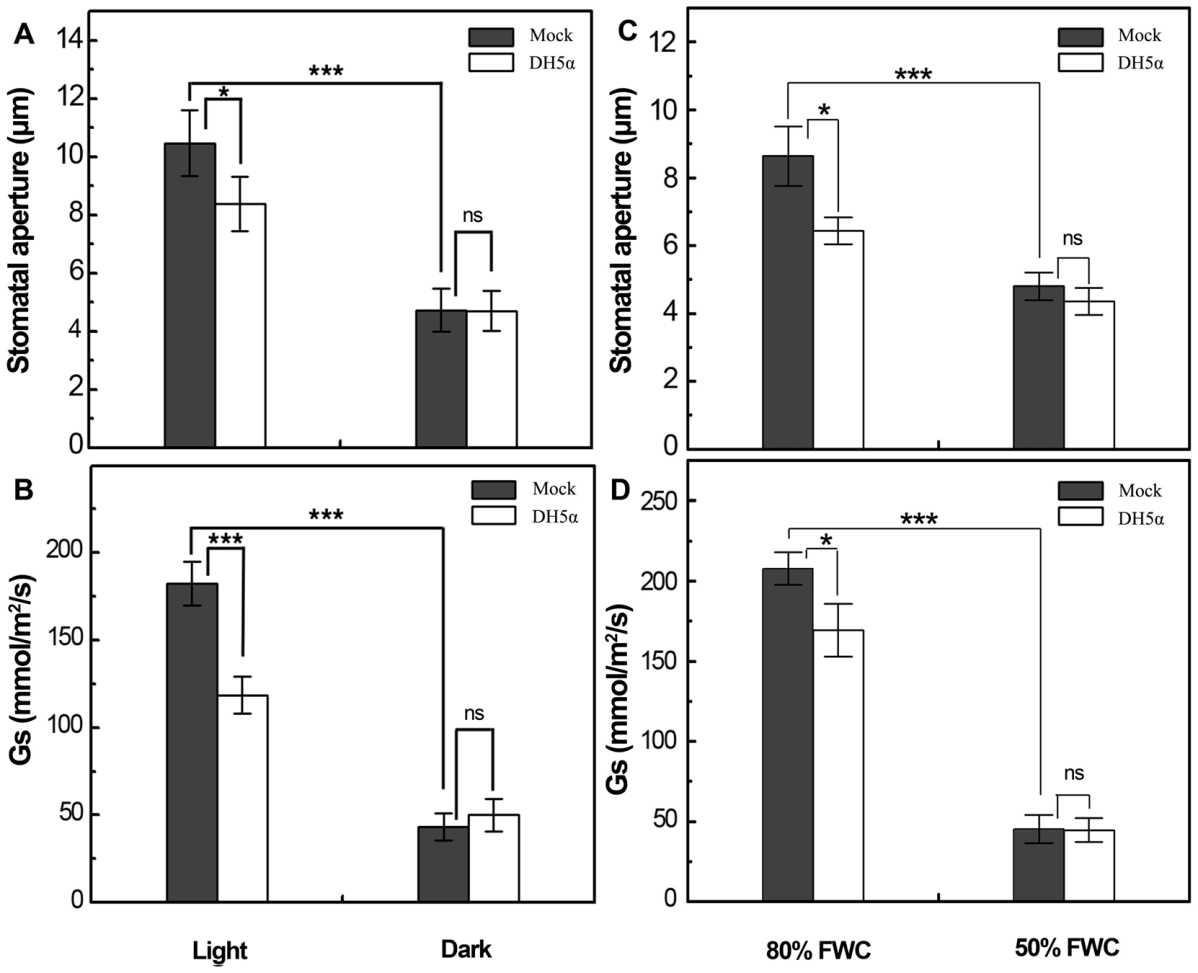
DH5α-triggered stomatal closure is disguised by darkness or drought treatment. A and B, Stomatal aperture and conductance in *V. faba* leaves dip-inoculated with mock or DH5α at 10^8^ CFU/ml under light/dark transition; C and D, Stomatal aperture and conductance in *V. faba* leaves dip-inoculated with mock or DH5α at 10^8^ CFU/ml under different field water content (FWC). Data from the epidermal bioassay are means of 120 stomatal aperture measurements from three replicates ±SEM. Data from the stomatal conductance experiment are means of measurements from 8–12 leaves (n = 4). Asterisks denote significant differences as analyzed by two-tailed *t*-test (***, *P*<0.001; *, *P*<0.05; ns, no statistical difference).

The above findings prompted us to ask if environmental signals inducing stomatal closure would also have effects on the stomatal immune response. It has been well established that darkness and root water deficiency can cause significant reduction in stomatal aperture [42], [43]. We therefore examined whether stomatal closure triggered by these two signals shows an additive effect on DH5α-induced stomatal closure in *Vicia faba*. It was found that darkness treatment completely removed the difference in stomatal aperture and Gs between mock- and DH5α-treated leaves (Figure 4A and 4B). In the root water starvation experiment, stomatal aperture and Gs in both mock- and DH5α-treated leaves reduced evidently when FWC dropped from 80% to 50% (Figure 4C and 4D). However, the stomatal pore were more closed in DH5α-treated leaves at 80% FWC when compared to that in the mock-treated ones. The differences in stomatal aperture and Gs between mock- and DH5α-treated leaves were completely disappeared when FWC reduced to 50%. Taken together, these data suggest that stomatal closing signals like darkness or drought can disguise stomatal closure elicited by DH5α bacteria.

### ABA can alleviate the aggressive multipliation of bacteria in the leaves under high relative humidity

**Figure 5:**
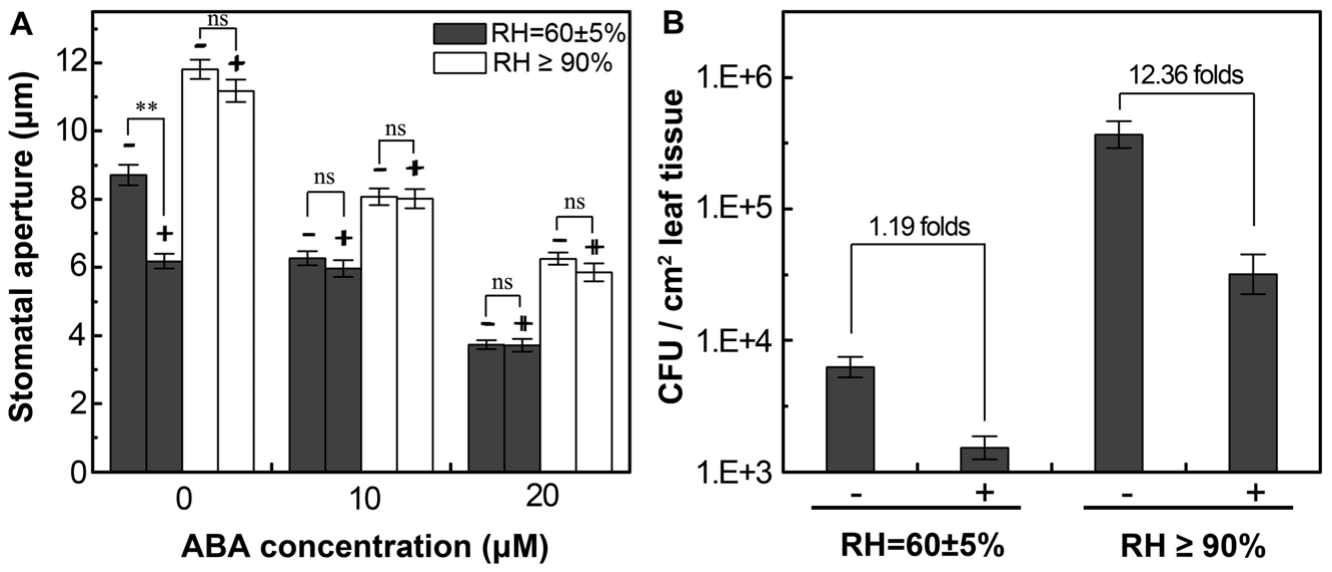
Exogenous ABA can reduce stomatal aperture and inhibit foliar bacterial growth under high RH. A, Stomatal aperture in *V. faba* leaves dip-inoculated with mock or DH5α (10^8^ CFU/ml) supplemented with the indicated concentrations of ABA under ambient and high RH conditions. Results represent means of three replicates ±SEM, (n = 120 stomata). “+” and “−” represent presence or absence of DH5α cells in the inoculum. Asterisks denote significant differences as analyzed by two-tailed *t*-test (**, *P*<0.01; ns, no statistical difference); B, Bacterial population in *V. faba* leaves at day 3 after dip inoculation with DH5α. “+” and “−” represent presence or absence of ABA (20 µM) in the inoculum.

The data above support the notion that stomata prioritize their response to multiple abiotic stimuli over the immune response to bacteria. Noticeably, DH5α-triggered stomatal closure in *Vicia faba* can be abolished under high RH (Figure 3C and 3D). Because of its biological significance in controlling the burst of bacterial diseases in the forest after heavy rains, we asked if stomatal closure under such a condition could be rescued by an imposed stomatal closing signal. To test this hypothesis, we dip inoculated faba bean plants pretreated under ambient or elevated RH, using the inoculum containing DH5α (10^8^ CFU/ml) and the indicated concentrations of ABA. It was found that, without exogenous ABA, DH5α induced stomatal closure in plants grown under ambient RH but not in those pretreated with high RH (Figure 5A). In contrast, a high concentration of ABA (10 or 20 µM) could reduce the stomatal aperture under both RH conditions (Figure 5A). It should be noted that there was no discernible additive effect between DH5α and ABA in the induction of stomatal closure (Figure 5A). We also determined DH5α counts in the faba bean leaves. It was observed that DH5α multiplied aggressively (about 146 folds) in the leaves pretreated under high RH compared to those grown under ambient RH (Figure 5B). Interestingly, application of exogenous ABA displayed a more profound inhibitory effect of bacterial growth in the leaves under high RH than in the ones grown under ambient RH (Figure 5B).

**Figure 6:**
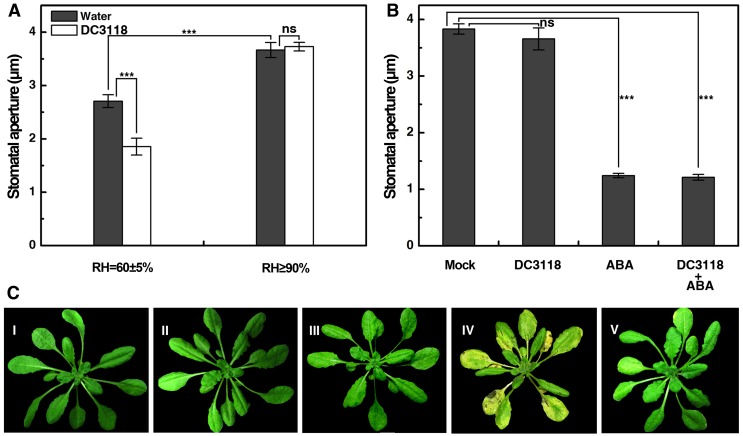
Pathogenicity of DC3118 in *Arabidopsis* can be modulated by extrinsic factors. A, Stomatal aperture in Col-0 leaves surface-inoculated with mock or DC3118 at 10^8^ CFU/ml under ambient or high RH; B, Stomatal aperture in Col-0 leaves surface-inoculated with different treatments under high RH. Data in A and B represent means of 120 stomatal aperture measurements from three replicates ±SEM. Asterisks denote significant differences as analyzed by two-tailed *t*-test (***, *P*<0.001; ns, no statistical difference); C, Progression of disease symptom in Col-0 plants with the following treatments: (I) RH = 60%, mock; (II) RH≥90%, mock; (III) RH = 60%, DC3118 (10^8^ CFU/ml); (IV) RH≥90%, DC3118 (10^8^ CFU/ml); (V) RH≥90%, DC3118 (10^8^ CFU/ml) + ABA (20 µM).

To see if the knowledge obtained from the DH5α-*Vicia faba* system can be applied during actual bacterial infection, we tested the pathogenicity of *Pst* DC3118 in wild-type (Col-0) *Arabidopsis* plants under ambient and high RH conditions with or without exogenous ABA. Biosynthesis of the predominant virulent factor coronatine is eliminated in this *P. syringae* strain and therefore it cannot infect plants displaying normal stomatal immune response [27]. We found that *Pst* DC3118 (10^8^ CFU/ml) significantly reduced the stomatal aperture under ambient RH, whilst high RH completely abolished such a response (Figure 6A). The disrupted stomatal closing response to *Pst* DC3118 under high RH could be restored by spraying a high level of ABA (20 µM) onto the leaves (Figure 6B). Consistently, *Pst* DC3118 did not induce any obvious leaf necrosis 4 days after inoculation under ambient RH (Figure 6C-II), whilst leaf necrosis was clearly discernible when plants inoculated with *Pst* DC3118 under high RH (Figure 6C-IV). Interestingly, the bacterial disease symptoms under high RH almost disappeared by application of exogenous ABA (Figure 6C-V). These data imply that pathogenicity of *Pst* DC3118 was restored under high RH that leads to stomatal opening, and this phenomenon might be due to impaired stomatal ABA signaling.

## Discussion

In the field, sessile plants are exposed to combinatorial, frequently changing environmental conditions that have profound effects on stomatal aperture. Plants also close their stomata actively as a means to prevent invasion of foliar pathogenic bacteria via the stomatal pore [26]–[29],[44]. In this study, we show that: (i) the lab use *E. coli* strain DH5α can trigger stomatal closure, which involves generation of ROS; (ii) environmental signals that induce stomatal opening abolish stomatal defense, whilst those promoting stomatal closure disguise the effect of bacteria on stomatal aperture; and (iii) multiplication of bacteria cells in plant leaves can be affected by manipulating abiotic signal inputs.

*E. coli* belongs to human pathogenic bacteria but also exists on the leaf surface of many plant species [45]–[47]. Previous studies have suggested that the recognition of pathogen associated molecular patterns (PAMPs) by receptors on the plant cell surface is the first step to initiate PTI [39], [48]. LPS is a lipopolysaccharide isolated from the cell wall of *E*. *coli*. Interestingly, we found that stomatal response to LPS is comparable to that elicited by DH5α, suggesting that *E. coli* might exploit LPS as an elicitor to trigger stomatal closure. Further studies using *E*. coli mutant defective in LPS biosynthesis or plant mutants lacking LPS receptors will provide direct evidence for this hypothesis. We also noticed that another recent study demonstrated the strong inductive effect of stomatal closure in *Arabidopsis* and lettuce by the human pathogenic *E. coli* strain O157:H7 even under high RH [49]. We reasoned that the apparently contradicting data might be a result of the difference in the *E. coli* strains used. In fact, compare to DH5α, the genome of O157:H7 contains 1387 new genes in strain-specific clusters of diverse sizes [50] (also see Table S1). These genes might encode putative elicitors conferring strain-specific abilities to induce stomatal closure even under favorable environmental conditions. Therefore, the difference in stomatal responses to DH5a and O157:H7 under high RH might be complicated, which needs to be clarified in future studies.

Generation of ROS is a critical signaling intermediate in PAMP-triggered stomatal closure [40]. In this work, we found that pretreatment of epidermal peels with DPI abolished DH5α-induced stomatal closure and H_2_O_2_ production in guard cells (Figure 2A and Figure S2), which suggests that NADPH oxidases might be responsible for the ROS elevation in the stomatal response to DH5a. In contrast, a previous study have shown that YEL-induced ROS accumulation in *Arabidopsis* guard cells can be eliminated by the peroxidase inhibitor salicylhydroxamic acid (SHAM) but not by DPI [40]. The previous findings, together with ours, suggest that plants may exploit distinct defensive mechanisms to prevent microbial entry via stomata.

Although how environmental stimuli affect stomatal defense still remains an open question, there are several lines of evidence suggesting that at least a subset of those effects might attribute to changes in the metabolism of plant hormones. ABA and SA have been shown to play critical roles in stomata-based immunity [27]–[29]. In this study, DC3118 was found to be non-pathogenic under ambient RH but become infective when RH increased to ≥90%. Additionally, foliar spraying of ABA restored stomatal defense against this bacterial strain at high humidity conditions (Figure 6). These data imply that the deficiency in stomatal immunity might be due to the aberrant ABA metabolism and/or signaling in leaves. Intriguingly, a recent study demonstrated that the activity of ABA 8′-hydroxylases, which catabolize or inactivate mobile ABA in guard cells and vascular tissues, is dramatically increased under high humidity conditions [25]. It will be interesting to check whether high humidity-abolished stomatal immunity can be restored, at least partially, in the null mutants of these enzymes.

Another possibility for how environmental stimuli affect stomatal immune response relies on the regulation of two mitogen-activated protein kinases, MPK3 and MPK6. Both of them have been proposed as “environmental sensors” that are able to perceive extrinsic signals and subsequently mediate an array of stress responses in plants [51]. Interestingly, MPK3 and MPK6 can be activated by the ROS signaling that is downstream of the flg22 perception at the cell surface [52]. It is reasonable to hypothesize that biotic and abiotic signals converge on MPK3/6 through two parallel pathways, and regulate stomatal aperture by fine-tuning the activities of both kinases.

In conclusion, we evaluated stomatal immune response under selected environmental conditions and found that stomata prioritize their opening or closing response when biotic and abiotic stimuli were imposed simultaneously. Stomatal responses to environmental signals tend to override that to microbial elicitors. Our work may help to provide a practical solution to control bacterial disease outbreaks in crops.

## Supporting Information

Figure S1 ***Pst*** **DC3118 can trigger stomatal closure in** ***V. faba*** **.** A. Stomatal aperture in *V. faba* epidermal peels incubated with DC3118 at the indicated concentrations; B. Stomatal aperture in *V. faba* epidermal peels incubated with mock or DC3118 at 10^8^ CFU/ml. Results represent means of three replicates ±SEM, (n = 120 stomata).
(TIF)

Figure S2 **DPI pretreatment eliminates DH5α-induced ROS accumulation in guard cells.** The microscopic images represent fluorescent and DIC images of peels treated with mock (upper left and right) and fluorescent and DIC images of peels inoculated with DH5α at 10^8^ CFU/ml (lower left and right). DPI (20 µM) was added to the opening buffer thirty minutes before H_2_DCF-DA loading. Bars  = 50 µm.
(TIF)

Table S1 **Comparison of features between DH5α and O157:H7.**
(DOCX)
