# Supplementary figures and images for: Stomata Prioritize Their Responses to Multiple Biotic and Abiotic Signal Inputs
Source: PLoS One. 2014 Jul 8;9(7):e101587. doi: 10.1371/journal.pone.0101587 (PMC4086820; doi:10.1371/journal.pone.0101587)

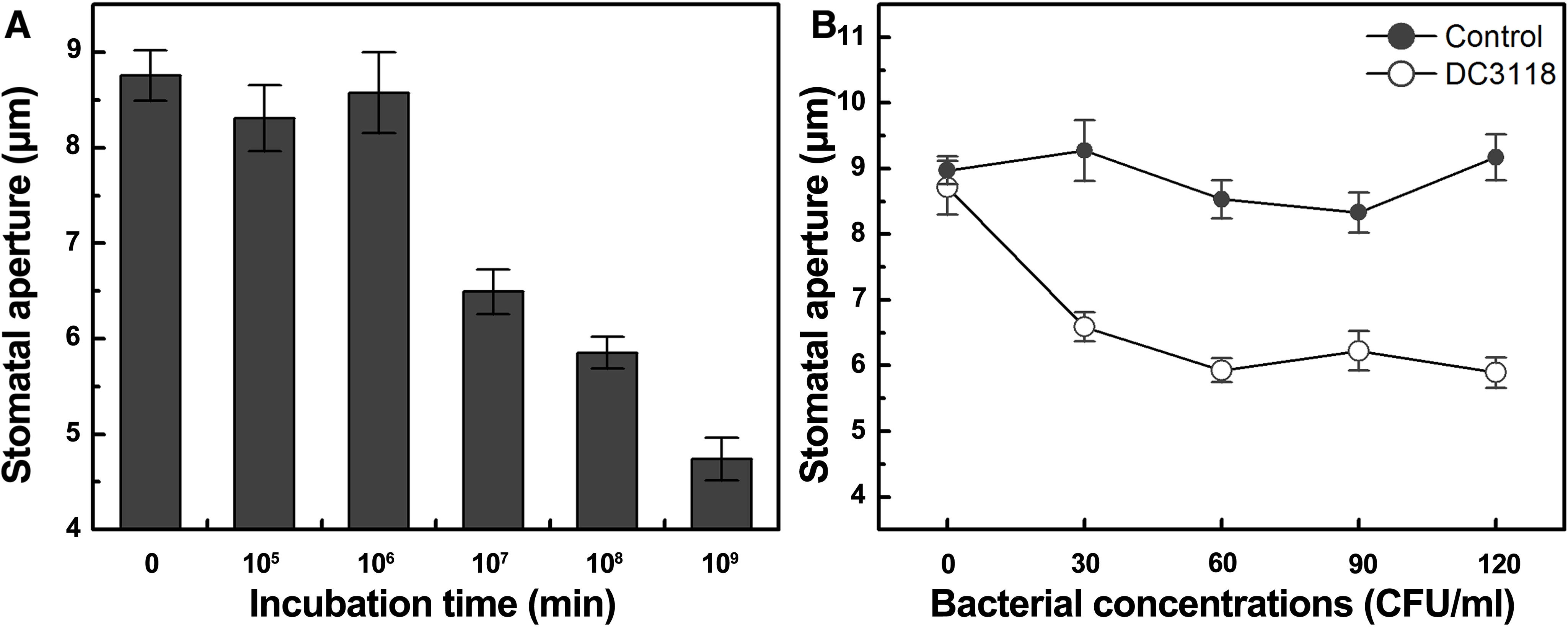

Supplement: Figure S1 — Pst DC3118 can trigger stomatal closure in V. faba . A. Stomatal aperture in V. faba epidermal peels incubated with DC3118 at the indicated concentrations; B. Stomatal aperture in V. faba epidermal peels incubated with mock or DC3118 at 108 CFU/ml. Results represent means of three replicates ±SEM, (n = 120 stomata). (TIF) [file pone.0101587.s001.tif]

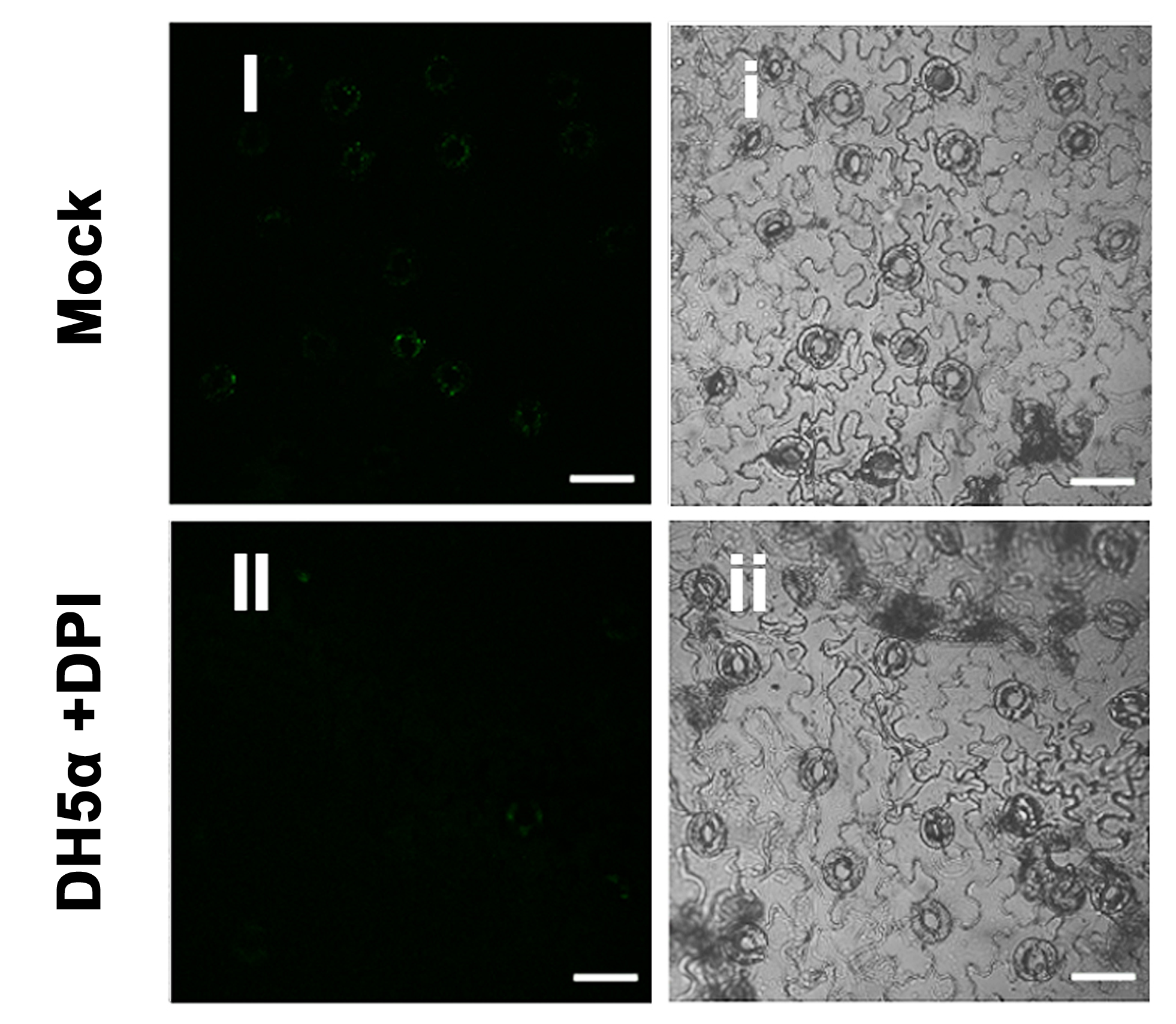

Supplement: Figure S2 — DPI pretreatment eliminates DH5α-induced ROS accumulation in guard cells. The microscopic images represent fluorescent and DIC images of peels treated with mock (upper left and right) and fluorescent and DIC images of peels inoculated with DH5α at 108 CFU/ml (lower left and right). DPI (20 µM) was added to the opening buffer thirty minutes before H2DCF-DA loading. Bars = 50 µm. (TIF) [file pone.0101587.s002.tif]
