# Supplementary material for: Stomata Prioritize Their Responses to Multiple Biotic and Abiotic Signal Inputs
Source: PLoS One. 2014 Jul 8;9(7):e101587. doi: 10.1371/journal.pone.0101587 (PMC4086820; doi:10.1371/journal.pone.0101587)
Supplement: Table S1 — Comparison of features between DH5α and O157:H7. (DOCX) [file pone.0101587.s003.docx]

**Table S1 Comparison of features between DH5α and O157:H7**

|  | **DH5a** | **O157:H7** |
| --- | --- | --- |
| **Possible elicitors** | LPS | LPS |
| **Virulent factors**  **in animals** | N/A | Verotoxin (shiga-like toxins like VT1 and VT2); TTSS; Adhesin; Heat stable enterotoxin; Heat labile enterotoxin. |
| **Bacterial strains** | K12 | O157:H7 |
| **Genic**  **features** | Mutant form of the K12 bacterial strain  [endA1](http://ecoliwiki.net/colipedia/index.php/endA:Gene): inactivates an intracellular endonuclease that degrades plasmid DNA in many miniprep methods.  hsdR17: eliminates the restriction endonuclease of the EcoKI restriction-modification system.  Δ(lacZ)M15: needed for blue-white screening with many lacZ based vectors.  recA: eliminates homologous recombination. This makes the strain somewhat sickly, but reduces deletion formation and plasmid multimerization.  glnV44: an [amber suppressor](http://ecoliwiki.net/colipedia/index.php/Nonsense_suppressor). | Compared to K12, the genome of O157:H7 contains 1387 new genes in strain-specific clusters of diverse sizes. These may encode candidate virulence factors, all of which could be targets for surveillance. Many clusters have no obvious role in virulence, but may confer strain-specific abilities to survive in different niches. |
